# Comparative Transcriptomics of Shiga Toxin-Producing and Commensal *Escherichia coli* and Cytokine Responses in Colonic Epithelial Cell Culture Infections

**DOI:** 10.3389/fcimb.2020.575630

**Published:** 2020-10-26

**Authors:** Lisa M. Harrison, David W. Lacher, Mark K. Mammel, Susan R. Leonard

**Affiliations:** Office of Applied Research and Safety Assessment, Center for Food Safety and Applied Nutrition, U.S. Food and Drug Administration, Laurel, MD, United States

**Keywords:** STEC, transcriptomics, cytokines, virulence factors, pathogenesis

## Abstract

Ingestion of Shiga toxin-producing *Escherichia coli* (STEC) can result in a range of illness severity from asymptomatic to hemorrhagic colitis and death; thus risk assessment of STEC strains for human pathogenicity is important in the area of food safety. Illness severity depends in part on the combination of virulence genes carried in the genome, which can vary between strains even of identical serotype. To better understand how core genes are regulated differently among strains and to identify possible novel STEC virulence gene candidates that could be added to the risk assessment repertoire, we used comparative transcriptomics to investigate global gene expression differences between two STEC strains associated with severe illness and a commensal *E. coli* strain during *in vitro* intestinal epithelial cell (IEC) infections. Additionally, we compared a wide array of concomitant cytokine levels produced by the IECs. The cytokine expression levels were examined for a pattern representing STEC pathogenicity; however, while one STEC strain appeared to elicit a proinflammatory response, infection by the other strain produced a pattern comparable to the commensal *E. coli*. This result may be explained by the significant differences in gene content and expression observed between the STEC strains. RNA-Seq analysis revealed considerable disparity in expression of genes in the arginine and tryptophan biosynthesis/import pathways between the STEC strains and the commensal *E. coli* strain, highlighting the important role some amino acids play in STEC colonization and survival. Contrasting differential expression patterns were observed for genes involved in respiration among the three strains suggesting that metabolic diversity is a strategy utilized to compete with resident microflora for successful colonization. Similar temporal expression results for known and putative virulence genes were observed in the STEC strains, revealing strategies used for survival prior to and after initial adherence to IECs. Additionally, three genes encoding hypothetical proteins located in mobile genetic elements were, after interrogation of a large set of *E. coli* genomes, determined to likely represent novel STEC virulence factors.

## Introduction

Shiga toxin-producing *Escherichia coli* (STEC) is a genomically diverse *E. coli* pathotype that produces Shiga toxin (Stx) and has been isolated from humans, animals, food, and many environmental sources (Garcia et al., 2010; Cooley et al., 2013). As a zoonotic foodborne pathogen, STEC primarily asymptomatically colonizes cattle; however, it has also been isolated from other domestic livestock and a variety of wild animals and birds (La Ragione et al., 2009; Cooley et al., 2013; Espinosa et al., 2018). Depending on their individual gene repertoire, STEC strains have the potential to be human pathogens associated with gastrointestinal illness, ranging from mild diarrhea to hemorrhagic colitis. In some cases, infection with STEC causes hemolytic uremic syndrome (HUS), a serious sequela that can progress to end-stage renal disease (ESRD) and lead to death (Kaper et al., 2004; Majowicz et al., 2014). Globally, based on data compiled between January 1990 and April 2012, there were an estimated 2.8 million illnesses associated with STEC, resulting in 3,890 cases of HUS, 270 cases of ESRD, and 230 deaths (Majowicz et al., 2014). Historically, STEC O157:H7 has been associated with outbreaks and severe illness (Kaper et al., 2004) and in 2018 and 2019 has been linked to multiple large outbreaks involving contaminated romaine lettuce (Centers for Disease Control and Prevention, 2020b). However, the proportion of non-O157 STEC illnesses has been increasing, and in fact, 58% of illnesses due to STEC infection were attributable to non-O157 serogroups in the US between 2008 and 2018 (Centers for Disease Control and Prevention, 2020a). Although at least 100 non-O157 serotypes have been known to cause human illness; those of serogroups O26, O45, O103, O111, O121, and O145 account for the greatest number of clinical cases (FAO/WHO STEC Expert Group, 2019; National Advisory Committee On Microbiological Criteria For Foods, 2019).

To cause illness, STEC must be ingested and survive passage through the gastrointestinal tract where it adheres to and colonizes the colon, followed by production of Stx. Prediction of the risk level as a human pathogen is important in the food industry since not all STEC strains cause illness in humans. STEC virulence genes are carried on mobile genetic elements, thus can be easily gained or lost. Therefore, while serotype is a useful indicator in epidemiological investigations, serotype alone is not considered a good predictor of clinical outcome. The best predictor of human pathogenicity is the set of virulence genes a particular STEC strain possesses, while also taking into consideration past illnesses linked with specific serotypes (Newell and La Ragione, 2018; FAO/WHO STEC Expert Group, 2019; National Advisory Committee On Microbiological Criteria For Foods, 2019). One virulence factor associated with severe clinical outcome is the type III secretion system (TTSS) encoded in the Locus of Enterocyte Effacement pathogenicity island (LEE PAI) that imparts, among other functions, the ability to adhere tightly to colonic intestinal epithelial cells (IECs) (Kaper et al., 2004). In risk assessments, the gene encoding the adhesin intimin, *eae*, is used as a marker for the presence of the LEE PAI. Enterohemolysin, encoded by *ehxA*, is also commonly considered when determining pathogenic potential, as it has been observed in many STEC strains causing severe disease (FAO/WHO STEC Expert Group, 2019; National Advisory Committee On Microbiological Criteria For Foods, 2019). However, there is not a perfect correlation between presence of *ehxA* and clinical outcome, which underscores the necessity of interrogating the genome for multiple factors that have been associated with virulence when determining risk, and this has become easier with the widespread use of whole genome sequencing (WGS) (Newell and La Ragione, 2018; Gonzalez-Escalona and Kase, 2019). Hybrid STEC pathotype strains have been increasingly reported (Leonard et al., 2016), the most important of which is the hybrid STEC/Enteroaggregative *E. coli* (EAEC) O104:H4 strain that caused a massive outbreak in 2011 with a high percentage of HUS (Rasko et al., 2011). EAEC carry *aggR*, encoding a transcriptional regulator of virulence genes including genes involved in adherence. The Stx subtype and presence/absence of *eae*, *ehxA*, and *aggR* are the principal virulence factors currently used in STEC risk assessments, although other adherence factors and toxins are taken into consideration. Progress on identification of novel STEC virulence genes has been slow; however, identification of novel STEC virulence factors is important since it would aid in refining suitable sets of genes to use in genome interrogations for risk determinations (Newell and La Ragione, 2018; FAO/WHO STEC Expert Group, 2019; National Advisory Committee On Microbiological Criteria For Foods, 2019). While WGS has been of great benefit to public health in the area of food safety (Allard et al., 2016), identification of additional STEC virulence factors by genome sequence comparisons alone remains elusive. For example, comparative genomics studies have revealed only previously identified virulence genes and were unable to differentiate STEC genomes by illness severity (Haugum et al., 2014; Baba et al., 2019).

In addition to the combination of virulence factors carried by an STEC strain, the dose ingested, and individual host responses can play a role in pathogenicity. Several reports have demonstrated that the interaction between the intestinal epithelium and different STEC factors, including lipopolysaccharides (Khan et al., 2006), flagellin (Berin et al., 2002; Rogers et al., 2003; Miyamoto et al., 2006), Stxs (Yamasaki et al., 1999; Thorpe et al., 2001), the TTSS factor EspT (Raymond et al., 2011), hemorrhagic coli pilus (HCP) (Ledesma et al., 2010), and long polar fimbriae (LPF) (Farfan et al., 2013; Vergara et al., 2014) can result in the induction of cytokines/chemokines that attract macrophages and polymorphonuclear leukocytes (PMNs) such as neutrophils *in vitro* and *in vivo*. Patients with STEC O157:H7 infections have been observed to possess high levels of PMNs in their feces (Slutsker et al., 1997), which is considered a possible risk marker of HUS development (Buteau et al., 2000). While most studies have focused on the induction of the neutrophil chemoattractant Interleukin-8 (IL-8), the induction of other cytokines and chemokines by intestinal epithelial cells (IECs) in response to STEC and its virulence factors has also been reported (Thorpe et al., 2001; Khan et al., 2006; Vergara et al., 2014; Stalb et al., 2018). Furthermore, depending on the experimental design, the cytokine profiles induced in various IEC models can vary greatly between different STEC strains and at times, but not always, from commensal *E. coli* (Hurley et al., 2001; Stalb et al., 2018). Comparison of induction levels of a wide variety of cytokines/chemokines by genetically diverse STECs known to have caused HUS and commensal *E. coli* may reveal patterns useful for predicting pathogenicity.

While STEC possesses virulence genes that are absent from commensal *E. coli* genomes, STEC and commensal strains share common core genes. Whether, and how, differences in global transcriptional responses of core genes contribute to human pathogenicity during survival and colonization in the gastrointestinal tract has not been elucidated. Previous reports have focused on either specific genes or global transcriptomic differences involving only one STEC O157:H7 strain adhered to epithelial cells (Jandu et al., 2009; Kim et al., 2009; Hu et al., 2013; Yang et al., 2015; Kumar and Sperandio, 2019). While these transcriptomic studies have been useful in determining regulation of select virulence genes and characterization of global responses in human infection, differences in transcriptional responses between STEC strains, and in contrast to commensal *E. coli*, upon adherence to IECs is unknown. Global transcriptomics studies involving sets of Enteropathogenic *E. coli* (EPEC) strains demonstrated phylogroup-specific responses to a variety of growth conditions but also individual differences between strains within the same phylogroup (Hazen et al., 2015; Hazen et al., 2017), thus highlighting the incomplete information gained from examining the transcriptional responses of only one strain in an *E. coli* pathotype.

In this study, we use an RNA-Seq approach to demonstrate differences and similarities among transcriptional responses of two non-O157 STEC strains associated with HUS that cluster with different phylogroups and a commensal *E. coli* strain in *in vitro* colonic IEC culture infections. In addition, we determine the concomitant cytokine/chemokine responses by the IECs, measuring an extensive panel of cytokine/chemokine levels in order to identify patterns that may be representative of pathogenic STEC. Through the RNA-Seq analysis combined with interrogation of a large set of *E. coli* genomes, we also identify hypothetical genes that represent potential novel STEC virulence factors.

## Materials and Methods

### Cell Culture

T84 cells (ATCC, Manassas, VA) were maintained at 37°C/5% CO_2_ in complete Dulbecco’s modified Eagle Medium/F12 (cDMEM/F12): DMEM/F12 (Invitrogen, Carlsbad, CA) supplemented with 10% heat-inactivated fetal bovine serum (FBS; Hyclone Laboratories, Logan, UT), 100 U/ml Penicillin/Streptomycin (Gibco, Gaithersburg, MD), and 2 mM L-glutamine (Gibco). T84 cells were passaged at no greater than 80% confluency. Caco-2 cells (ATCC) were maintained at 37°C/5% CO_2_ in complete DMEM (cDMEM): low glucose DMEM (Invitrogen) supplemented with 10% heat-inactivated FBS, 100 U/ml Penicillin/Streptomycin, and 2 mM L-glutamine.

### Bacterial Isolates and Growth Conditions

Two STEC strains and one *E. coli* strain known to be commensal were used in this study. Draft whole genome sequences for STEC O26:H11 strain 97-3250 and STEC O145:H28 strain 4865/96 are publicly available with accession numbers JHEW01000000 and JHEY01000000, respectively. Annotation of the genomes was provided by NCBI. The genome sequence of the commensal *E. coli* O9:H4 strain HS is also publicly available (accession no. CP000802.1). For planktonic cultures, overnight cultures of the *E. coli* were grown in Luria broth at 37°C and used in subcultures at a ratio of 1:100 into 25 ml DMEM/F12 in a 250 ml flask. The flasks were shaken at 185 rpm and 37°C until the cultures were in late exponential growth phase. RNAprotect Bacteria Reagent (Qiagen, Germantown, MD) was used on aliquots of the planktonic cultures and pellets were stored at −80°C for RNA extraction. Biological triplicate cultures were grown for use in the RNA-Seq analysis. *E. coli* cultures were grown for use in *in vitro* infection experiments with polarized T84 IEC cultures using the same method as for planktonic cultures, except that to simulate passage through the small intestine, DMEM/F12 was supplemented with 0.4% w/v porcine bile extract (Sigma, St. Louis, MO). To obtain the correct density of *E. coli* cells for the infection experiments, aliquots of cultures were pelleted and resuspended in 250 µl of media as needed to yield a multiplicity of infection of 100:1 when added to the IECs. For STEC cultures to be used for both planktonic and adhered transcriptomics experiments using polarized Caco-2 IECs, the same procedure was used as for the T84 IEC experiments with the exception that low glucose DMEM was substituted for DMEM/F12.

### T84 Cell Infection

T84 cells (1 × 10^6^ cells/well), at passage >8, were seeded onto 6-well tissue culture plates containing collagen-coated (5 μg/cm^2^) transwell inserts (Corning, Lowell, MA). Media was changed every 2–3 days until transepithelial electrical resistance (TEER) measurements were at least 1,000 Ω (approximately 14 days) as measured by the EVOM2 Epithelial Voltohmmeter (World Precision Instruments, Sarasota, FL). *E. coli* cells were added in a volume of 250 μl into 1.75 ml of fresh cDMEM/F12 in the transwell insert. The plates were rocked back and forth and side to side to distribute bacteria evenly within the transwell insert. Each plate was centrifuged at 3,000 rpm for 1 min at RT and incubated at 37°C/5% CO_2_ for 3 h. Basolateral supernatants were collected and centrifuged at 14,000 rpm for 2 min at 4°C. Aliquots of basolateral supernatants were transferred to new microcentrifuge tubes and stored at −80°C for cytokine analysis. T84 cells and adherent bacteria remaining in the transwell inserts were rinsed two times with sterile 1× phosphate buffered saline (PBS) to remove residual non-adherent bacteria. To each transwell, 1 ml of RLT lysis buffer (RLT buffer containing *β*-mercaptoethanol) from the RNeasy mini kit (Qiagen) was added, and the adherent cells were gently scraped from the membrane using a sterile cell lifter (Corning). The contents from two transwells were combined for a total of 2 ml, vortexed 1 min, and centrifuged at 14,000 rpm for 2 min at 4°C to pellet adherent bacterial cells. Supernatants, which contained the contents of the lysed T84 cells, were transferred to new microcentrifuge tube and homogenized using 1 cc VanishPoint Syringes with 25 G × 5/8″ needles (Retractable Technologies, Inc, Little Elm, Texas). The remaining pellets of adherent bacterial cells were immediately resuspended in 300 µl PBS followed by the use of RNAprotect Bacteria Reagent to stabilize the RNA and stored at −80°C for RNA extraction. Biological triplicate infection experiments were performed starting with separate overnight cultures for each of the three *E. coli* strains.

### Caco-2 Cell Infection

Caco-2 cells (0.5 × 10^6^ cells/well), at passage >70, were seeded onto 6-well tissue culture plates (Corning). Media was changed every 2–3 days until TEER measurements were at least 400 Ω (approximately 21 days) as measured by the EVOM2 Epithelial Voltohmmeter. Three-hour infection experiments were performed in triplicate using the STEC strains. The same procedure was followed as for the T84 cell infections with the exception that low glucose DMEM media was used.

### Bacterial RNA Isolation and Sequencing

Total RNA was extracted from both planktonic and adhered *E. coli* cell pellets using the RNeasy Mini Kit (Qiagen). The RNA was treated twice with DNase I from the Turbo DNA-free Kit (Ambion, Austin, TX) to remove contaminating DNA, followed by a clean-up step using the RNeasy Mini Kit. Depletion of bacterial rRNA from total RNA samples was accomplished using the MICROB*Express* Bacterial mRNA Enrichment Kit (Ambion), after which RNA quality was assessed using a Bioanalyzer (Agilent, Santa Clara, CA). Sequencing libraries were constructed from the mRNA-enriched RNA samples using the TruSeq RNA Library Prep Kit v2 (Illumina, San Diego, CA), utilizing the protocol modifications as designated by the manufacturer for bacterial RNA. The libraries were sequenced using 75 bp paired-end sequencing on an Illumina MiSeq platform generating an average of 17.8 million reads per sample.

### RNA-Seq Analysis

RNA-Seq analysis was performed using CLC Genomics Workbench version 12.0 (https://digitalinsights.qiagen.com) with default parameters. The sequence reads generated for each of the biological triplicate planktonic and adhered bacterial RNA samples were trimmed for quality, and adapter sequences were removed. The trimmed reads were mapped to the respective *E. coli* genomes imported as GenBank files, thus using the open reading frame annotations provided by NCBI. Differential expressions were calculated for adhered bacterial cells compared to planktonic bacterial cells for each *E. coli* strain, along with statistical analysis using the triplicate samples for each condition. CLC Genomics Workbench uses the normalization process to correct for sample library size used in edgeR (Robinson et al., 2010), and the False Discovery Rate (FDR) corrected *p* values were determined using the method of Benjamini and Hochberg. Differentially expressed genes were defined as those with fold adhered/planktonic changes of either >2 or <−2 and FDR (corrected *p* value) <0.05. The differential expression results were exported, and gene homolog results were used to compare differential expression for adhered *versus* planktonic cultures among the three *E. coli* strains.

### *E. coli* Genome Comparisons

Shared genes were identified in the three *E. coli* genomes by pairwise megablast comparisons using a custom Python program. Genes were considered homologs if ≥90% of the gene length had ≥90% nucleotide identity and were reciprocal best matches. Amino acid sequences were used to assign genes to biochemical pathways in the KEGG database. To determine which *E. coli* genomes possess selected hypothetical genes found in the two STEC genomes, a megablast search of a database containing 25,527 publicly available *E. coli* genomes as well as *Escherichia* cryptic lineage 1 and 6 (the two cryptic lineages that cluster most closely with *E. coli*) was performed. Parameters used to determine gene presence were at least 90% similarity over at least 67% of the query length. *E. coli* genomes representing the entire *E. coli* genomic landscape were used, thus included genomes both with and without known molecular markers representing the *E. coli* pathotypes. The genomes were classified as belonging to one or more of six pathotypes, namely, Attaching and Effacing *E. coli* (AEEC), EAEC, Enteroinvasive *E. coli* (EIEC), Enterotoxigenic *E. coli* (ETEC), Extraintestinal pathogenic *E. coli* (ExPEC), and STEC. The pathotypes were defined as carrying one or more of the following molecular markers: AEEC (*eae*), EAEC (aggR*, aafA*), EIEC (*ipaH, icsP, mkaD, ospB*), ETEC (*LT, ST*), ExPEC (*papD, afaD, cnf, hlyA*), STEC (*stx1, stx2*). Genomes not falling into one or more of these pathotypes were classified as “other”.

### Human Cytokine Analysis

Cell-free basolateral T84 supernatants were analyzed in duplicate using the Bio-Plex Pro™ Human Chemokine Panel, 40-Plex Kit (#171AK99MR2; Bio-Rad, Hercules, CA), and the Bio-Plex 200 Multiplex Reader (Bio-Rad), according to the manufacturer’s instructions. Concentration in range values were used for analysis to identify differences in cytokine expression among the uninfected and infected T84 cells. Samples from three independent experiments were analyzed in duplicate and graphed using GraphPad Prism 6 (GraphPad Software, La Jolla, CA). For samples where every value was out of range, indicating very low levels of cytokine production, a value of 0.1 was substituted for statistical analysis. The data are expressed as the means of the concentrations (pg/ml) ± standard errors of the mean (SEM). Statistical significance was assessed at a *p* value of <0.05 by one-way ANOVA with Tukey’s multiple comparisons test using Graphpad Prism 6.

## Results

### Gene Homologs in the *E. coli* Strains

**Figure 1:**
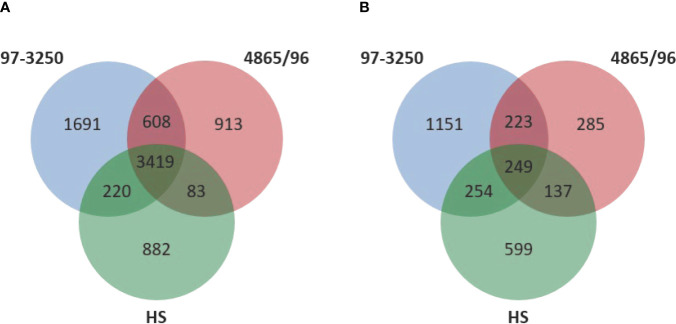
Comparison of genomic content and differentially expressed genes in the STEC strains and commensal *E. coli* HS. **(A)** Venn diagram comparing the number of shared and unique genes among the two STEC and HS strains. DNA homology of open reading frames among the strains was used to determine gene homologs. The rRNA genes are not included. **(B)** Venn diagram comparing the number of shared and unique differentially expressed genes for adhered compared to planktonic conditions among the STEC and HS strains.

Two non-O157 STEC strains and one commensal *E. coli* strain were selected for this study. Both STEC strains, STEC O26:H11 strain 97-3250, and STEC O145:H28 strain 4865/96, are LEE-positive strains with a known association with HUS. However, the two STEC strains belong to different *E. coli* phylogroups. STEC 97-3250 clusters with phylogroup B1, while STEC O145:H28 clusters with phylogroup E. The commensal *E. coli* strain HS belonging to phylogroup A was used for comparison. To enable transcriptional response comparisons among the strains, gene homologs were identified in the genomes of the three strains using megablast matching of nucleotide sequences of annotated open reading frames in the genomes (**Figure 1A**). The number of genes, after removal of rRNA genes, is 5,938, 5,023, and 4,604 for strains 97-3250, 4865/96, and HS, respectively (**Table 1**). Of these, 3,419 genes are shared between all three strains and 608 genes are shared between the two STEC strains, exclusive of HS. The genome of each strain contains over 800 unique genes.

**Table 1:** Number of genes in the *E. coli* genomes and genes differentially expressed in adhered compared to planktonic conditions.

*E. coli* strain	Genes^1^	DEGs^2^ (up/down)
STEC O26:H11 strain 97-3250	5,938	1,877 (950/927)
STEC O145:H28 strain 4865/96	5,023	894 (486/408)
*E. coli* O9:H4 strain HS	4,604	1,239 (688/551)

^1^does not include rRNA genes.

^2^differentially expressed genes.

### Global Transcriptional Responses

**Figure 2:**
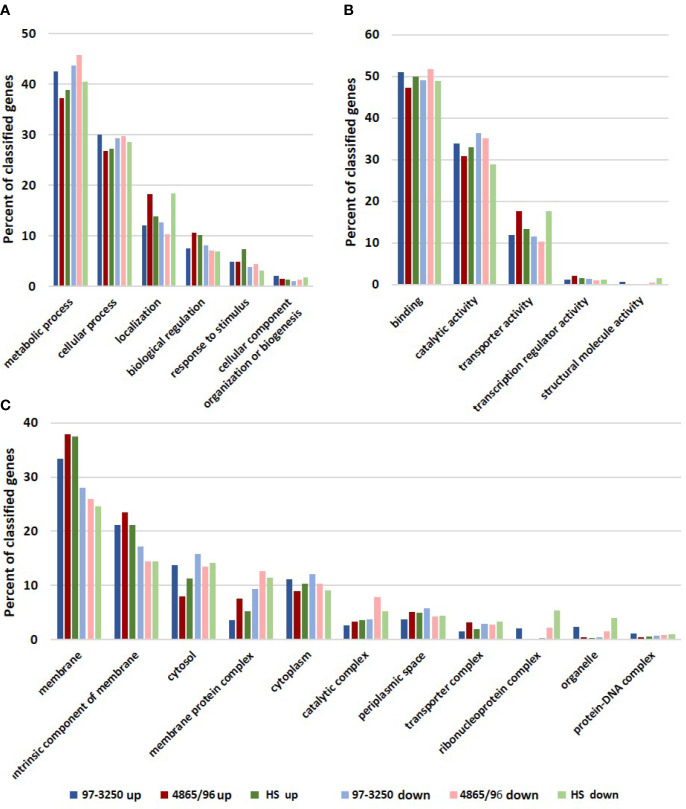
Biological functions of differentially expressed genes. Genes differentially expressed by each of the STEC or commensal *E. coli* adhered to T84 IECs compared to planktonic culture were classified using the Gene Ontology (GO) database. The number of upregulated and downregulated genes classified in each category were used to determine percentages in subcategories within the three main categories in the GO database: **(A)** biological process, **(B)** molecular function, and **(C)** cellular component.

The STEC and commensal *E. coli* strains were used in *in vitro* infection experiments with polarized T84 colonic IECs. Genes within the LEE PAI are expressed during planktonic growth in virulence-promoting conditions as used in this study and found to be most highly expressed in late exponential growth phase but decreasing after adherence to IECs (Knutton et al., 1997; Hazen et al., 2015; Yang et al., 2015). We reasoned that some other virulence genes are also likely to exhibit greater expression differences between these two conditions. Thus, to maximize the possibility of discovering novel virulence genes in the STEC strains, we utilized RNA-Seq to compare *E. coli* transcriptomic results between late exponential planktonic growth and 3 h after infection with IECs. The global transcriptomes were determined for each of the three strains for both conditions, and significantly differentially expressed genes (DEGs) in adhered *E. coli* compared to planktonic *E. coli* were identified. Gene homolog matches among the three strains were used to determine shared DEGs (**Figure 1B**). RNA-Seq analysis identified 472 DEGs shared between the STEC strains, and of those, 76 do not have a homolog in HS. The HS genome does carry a homolog for the remaining 396 DEGs, but only 249 of those genes are also differentially expressed in HS. The number of DEGs for each strain were 1,877 (31.6%), 894 (17.8%), and 1,239 (26.9%) for strains 97-3250, 4865/96, and HS, respectively (**Table 1**). For all three strains, the number of upregulated genes was higher than the downregulated genes, but the difference was small, particularly in 97-3250 where it was only 23 genes. Variability in expression patterns for adhered compared to planktonic *E. coli* was observed among the strains. Of the 249 DEGs shared among all three strains, not all exhibited a fold change in the same direction and/or with similar magnitude. For some genes, disparity was observed between the transcriptional responses of the two STEC strains, and in others, the two STECs displayed a similar response that was different than that observed for HS. To determine the main biological functions of the DEGs, the genes were mapped to terms in the Gene Ontology (GO) database. For genes that could be classified, the percentage of DEGs was determined for each of the three main categories: biological process (**Figure 2A**), molecular function (**Figure 2B**), and cellular component (**Figure 2C**). In general, the percentages for the classifications in the three strains were similar, with metabolic process, cellular process, and localization having the highest percentages in the biological process category and binding, catalytic activity, and transporter activity in the molecular function category. Interestingly, for two of the subcategories, namely, localization within biological process and transporter activity within molecular function, there was a greater percentage of upregulated compared to downregulated genes for 4865/96, while the reverse was observed for HS. The greatest percentage of DEGs in the cellular component category was classified as membrane and intrinsic component of the membrane, and there was a higher percentage of upregulated compared to downregulated DEGs.

### Selected Biochemical Pathways and Genes Exhibiting Different Expression Patterns Between the STEC Strains and the Commensal *E. coli* Strain

**Figure 3:**
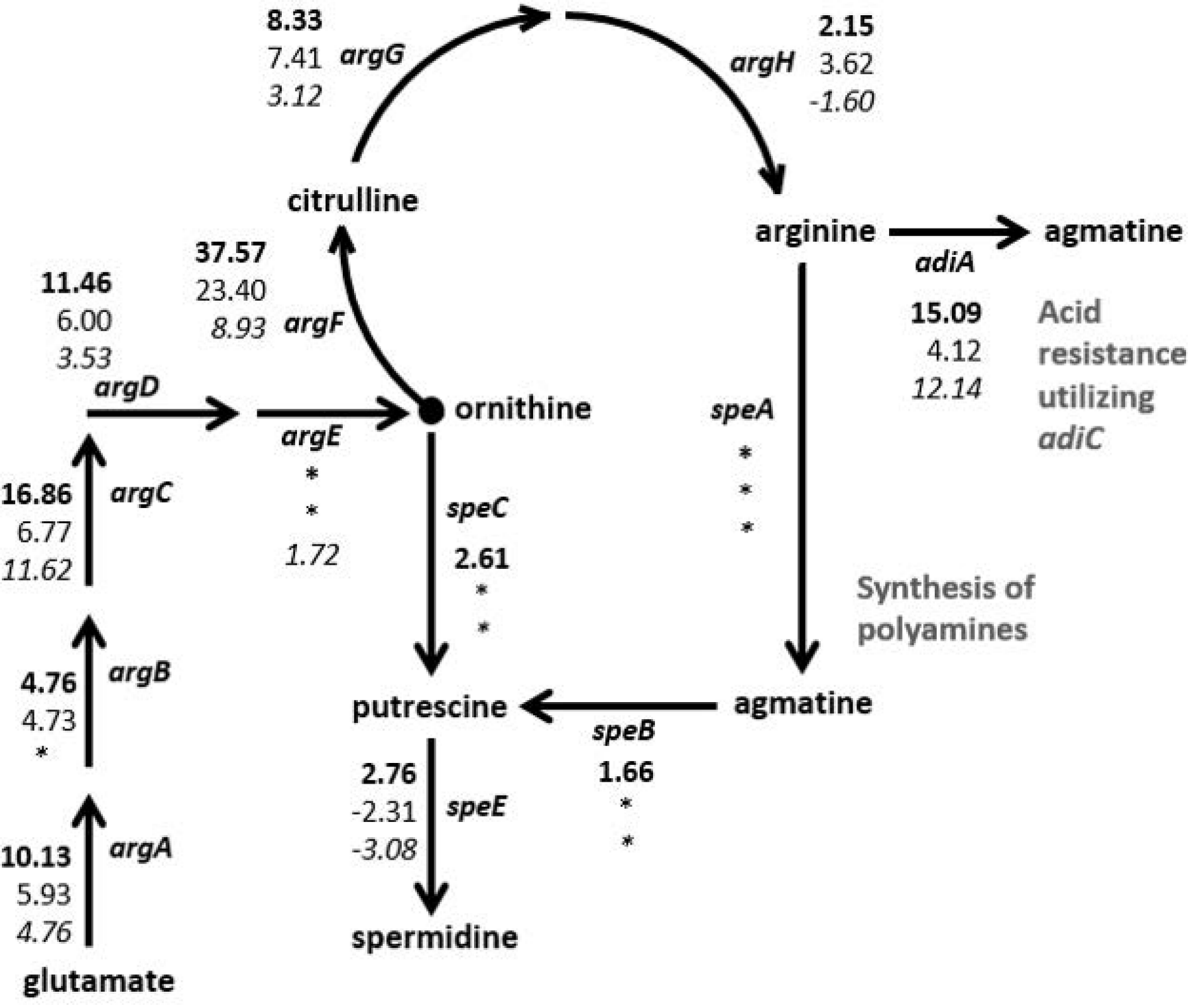
Differential expression of genes in the arginine and polyamine biosynthesis pathway in *E. coli*. Fold changes between the adhered and planktonic states for genes in the biosynthesis of arginine and polyamines were determined and are indicated next to the respective gene in the diagram. Numerical values for which the FDR adjusted *p* value < 0.05 are included, otherwise an asterisk is included instead. Fold change values are listed in descending order for strains 97-3250 (bold font), 4865/96 (standard font), and HS (italic font).

The RNA-Seq results were examined for genes that were regulated similarly in the STEC strains but either in the opposite direction than observed for HS or to a dissimilar extent. In particular, two biochemical pathways, the arginine biosynthesis/import and tryptophan biosynthesis/import pathways, demonstrated considerable disparity in differential expression patterns between the two STECs and the commensal HS (**Figure 3**, **Table 2**). Nearly all of the genes involved in arginine biosynthesis were more highly transcribed in adhered *E. coli* cells compared to planktonic cultures in the STEC strains compared to HS. In addition, the *art* genes, involved in importing arginine from the environment, and *arcD*, the gene encoding the arginine:ornithine antiporter that concurrently exports ornithine while importing arginine were more highly transcribed in the adhered compared to planktonic STEC cells in contrast to HS. However, *argR*, encoding the arginine repressor, was not differentially expressed. The polyamine putrescine can be synthesized from ornithine or agmatine (**Figure 3**) as well as imported from the environment. The transcription of genes involved in the conversion of arginine or ornithine to putrescine is unchanged in 4865/96 and HS, and *speE*, encoding the enzyme required for synthesis of spermidine from putrescine, is downregulated. However, the genes involved in the synthesis of putrescine and spermidine are upregulated in 97-3250. Importantly, although the transcription of genes used for the synthesis of polyamines is unchanged in HS, import of polyamines utilizing both the importers encoded by *yeeF* and the *pot* genes is downregulated, while it is upregulated in 97-3250 and either unchanged or downregulated to a lesser extent in 4865/96 than in HS. The tryptophan biosynthesis pathway was dramatically downregulated in adhered HS compared to planktonic culture, while for both STEC strains, transcription of the genes in the pathway was either increased, unchanged, or in the case of *trpA*, decreased but to a much lesser extent than observed for HS (**Table 2**). Transcription of *mtr*, encoding a tryptophan importer, was also significantly decreased in adhered HS, but increased in both STECs. Additionally, transport of other biomolecules was regulated differently in the STECs compared to HS. Dipeptide transport using the product of the *dpp* genes, as well as expression of *ompF*, was downregulated to a greater extent in HS in comparison to the STECs. The *mgtA* gene, encoding a transport protein mediating import of magnesium, was more highly expressed in adhered STEC compared to planktonic culture, while expression was unchanged in HS. Pronounced disparity in transcriptional responses between the STECs and HS were also observed for the *pstSCAB-phoU* operon and the two-component regulatory system encoded by *phoBR* that control the phosphate regulon. Aspartate kinase catalyzes the first reaction in the aspartate pathway in which aspartic acid is metabolized to produce the amino acids lysine, threonine, methionine, and isoleucine. The gene encoding aspartate kinase, *thrA*, was downregulated in the adhered STEC strains, while not differentially expressed in HS.

**Table 2:** Selected biochemical pathways or genes exhibiting similar differential expression patterns for adhered compared to planktonic conditions in the STEC strains and in contrast to the commensal HS strain.

Gene	Function	Fold change 97-3250	Fold change 4865/96	Fold change HS
*artJ*	Arginine transporter (uptake)	11.25	10.30	8.94
*artM*	Arginine transporter (uptake)	2.90	3.49	−2.22
*artQ*	Arginine transporter (uptake)	2.42	2.64	−1.89
*artI*	Arginine transporter (uptake)	1.83	NS	−2.03
*artP*	Arginine transporter (uptake)	3.40	3.92	NS
*arcD*	Arginine:ornithine antiporter	7.69	2.01	NS
*adiC*	Arginine:agmatine antiporter	20.58	53.51	33.29
*yeeF*	Putrescine importer	4.48	−2.53	−6.81
*potA*	Putrescine and spermidine import	3.05	NS	−1.89
*potB*	Putrescine and spermidine import	3.57	NS	−3.34
*potC*	Putrescine and spermidine import	2.33	NS	−3.36
*potD*	Putrescine and spermidine import	−1.60	−2.83	−6.42
*puuA*	Synthesis of glutamate from putrescine	5.23	5.64	NS
*trpA*	Tryptophan biosynthesis	−3.34	NS	−98.96
*trpB*	Tryptophan biosynthesis	NS	NS	−99.23
*trpC*	Tryptophan biosynthesis	NS	NS	−44.56
*trpD*	Tryptophan biosynthesis	3.82	2.21	−68.24
*trpE*	Tryptophan biosynthesis	10.86	9.71	−135.51
*mtr*	Tryptophan transport	3.98	2.91	−23.20
*tnaA*	Synthesis of indole from tryptophan	−4.99	−7.75	NS
*dppA*	Dipeptide transport and peptide chemotaxis	−5.46	−1.78	−5.74
*dppB*	Dipeptide transport and peptide chemotaxis	−2.29	−2.09	−11.86
*dppC*	Dipeptide transport and peptide chemotaxis	−2.96	−2.37	−26.14
*dppD*	Dipeptide transport and peptide chemotaxis	−4.69	−2.90	−27.34
*dppF*	Dipeptide transport and peptide chemotaxis	−3.68	−3.38	−21.86
*mgtA*	Magnesium import into cytosol	13.51	6.32	NS
*ompF*	Transport porin regulating osmotic pressure	−14.77	−15.25	−30.85
*phoU*	Negative regulator of the Pho regulon	−3.08	−3.54	−33.63
*phoB*	Response to extracellular phosphate concentration	1.92	NS	−10.84
*phoR*	Response to extracellular phosphate concentration	NS	NS	−15.32
*phoA*	Alkaline phosphatase (dephosphorylation in the periplasm)	−2.86	−2.35	−68.12
*pstA*	Transport of inorganic phosphate and negative regulation of the Pho regulon	NS	NS	−44.88
*pstB*	Transport of inorganic phosphate and negative regulation of the Pho regulon	−1.58	−2.92	−34.43
*pstC*	Transport of inorganic phosphate and negative regulation of the Pho regulon	NS	NS	−38.80
*pstS*	Transport of inorganic phosphate and negative regulation of the Pho regulon	NS	NS	−80.92
*thrA*	Phosphorylation of aspartate	−4.08	−5.89	NS

NS, not statistically significant (FDR > 0.05).

### Contrasting Expression Patterns Were Observed for Genes Involved in Respiration

Although all three *E. coli* strains displayed marked differences in the use of respiratory pathways when converting from a planktonic to an adhered lifestyle, there was variability in gene expression changes associated with aerobic and anerobic respiration among them (**Table 3**). Contrasting transcriptional responses were observed among the three strains for genes encoding the transcription factors *fnr* and *arcA* that facilitate metabolic shifts between aerobic and anaerobic conditions (Lin and Iuchi, 1991; Jiang et al., 2015). While there were genes or sets of genes encoding subunits of larger proteins that were regulated similarly in the STECs, but different than HS, overall there was no distinguishing transcriptional pattern differentiating the STEC strains from HS. For example, all of the *nuo* genes that encode the NADH ubiquinone oxidoreductase respiratory complex I involved in aerobic respiration were downregulated to a greater extent in HS than observed for the STEC strains. However, transcription of cytochrome oxidase, encoded by *cyoABCD*, was decreased to a greater extent in 4865/96 and HS than in 97-3250. Genes involved in anaerobic respiration utilizing, for example, glycerol, lactate, and formate are regulated to different magnitudes among the strains under the two conditions tested. At 3 h post infection, expression of the genes encoding formate hydrogenlyase, which is involved in anaerobic oxidation of formate, was considerably higher in 4865/96 and HS, but not differentially expressed in 97-3250. Expression of the *glpABC* operon, encoding the glycerol 3-phophate dehydrogenase utilized in anaerobic respiration was downregulated in both STECs, while not in HS. In contrast, *glpD*, involved in aerobic respiration, was upregulated in 97-3250 and HS, but not 4865/96. Additionally, the DEGs, *narG* and *napA*, involved in anaerobic nitrate respiration were transcribed with considerable differences between the STECs and HS. In general, the fold change values exhibited by 4865/96 and HS were greater in magnitude, suggesting a greater shift in metabolic pathways used for respiration between planktonic growth and adhered cells than for 97-3250.

**Table 3:** Differential expression of *E. coli* genes involved in respiration for adhered compared to planktonic conditions.

Gene	Function	Fold change 97-3250	Fold change 4865/96	Fold change HS
arcA	aerobic respiration control	NS	NS	2.40
arcB	aerobic respiration control sensor	−2.13	NS	1.72
Fnr	fumarate and nitrate reduction regulatory protein	3.74	NS	NS
cyoA	cytochrome o ubiquinol oxidase subunit	−5.34	−5.75	−4.23
cyoB	cytochrome o ubiquinol oxidase subunit	−4.65	−16.04	−12.83
cyoC	cytochrome o ubiquinol oxidase subunit	−1.94	−24.24	−16.22
cyoD	cytochrome o ubiquinol oxidase subunit	NS	−52.90	−28.91
fumA	fumarate hydratase	NS	v3.36	−5.69
glpD	glycerol-3-phosphate dehydrogenase	7.09	NS	4.84
maeB	malate dehydrogenase	−3.03	−3.30	1.98
acsA	acetyl-CoA synthetase	2.04	−2.97	−4.97
nuoA	NADH:ubiquinone oxidoreductase subunit	NS	−2.14	−3.47
nuoB	NADH:ubiquinone oxidoreductase subunit	−1.80	−3.17	−4.48
nuoC	NADH:ubiquinone oxidoreductase subunit	NS	−3.44	−5.02
nuoE	NADH:ubiquinone oxidoreductase subunit	−2.34	−3.90	−6.68
nuoF	NADH:ubiquinone oxidoreductase subunit	−2.36	−3.82	−7.77
nuoG	NADH:ubiquinone oxidoreductase subunit	−2.14	−3.26	−6.17
nuoH	NADH:ubiquinone oxidoreductase subunit	−2.29	−2.69	−7.36
nuoI	NADH:ubiquinone oxidoreductase subunit	−2.70	−3.04	−6.05
nuoJ	NADH:ubiquinone oxidoreductase subunit	−2.53	−2.61	−7.01
nuoK	NADH:ubiquinone oxidoreductase subunit	−2.75	−2.66	−9.13
nuoL	NADH:ubiquinone oxidoreductase subunit	−2.79	−2.43	−6.93
nuoM	NADH:ubiquinone oxidoreductase subunit	−2.47	−2.79	−5.06
nuoN	NADH:ubiquinone oxidoreductase subunit	−1.99	−2.46	−4.90
cydA	cytochrome d ubiquinol oxidase subunit	NS	NS	NS
cydB	cytochrome d ubiquinol oxidase subunit	NS	NS	NS
narG	Anaerobic nitrate respiration	2.30	NS	23.23
narZ	Nitrate reductase	2.22	3.83	2.31
napA	Anaerobic nitrate respiration	−3.38	−4.26	−25.12
dmsA	Reduction of DMSO during anaerobic respiration	−5.79	−8.94	NS
dmsB	Reduction of DMSO during anaerobic respiration	−5.17	−5.03	NS
dmsC	Reduction of DMSO during anaerobic respiration	−3.60	NS	NS
glpA	Anaerobic glycerol 3-phophate dehydrogenase	−5.94	−26.77	NS
glpB	Anaerobic glycerol 3-phophate dehydrogenase	−4.62	−14.54	NS
glpC	Anaerobic glycerol 3-phophate dehydrogenase	−3.10	−8.37	NS
adhE	acetaldehyde dehydrogenase	−7.97	NS	3.78
ackA	Acetate kinase	NS	2.34	NS
ndh	NADH dehydrogenase II	3.77	5.48	2.61
aceE	Pyruvate dehydrogenase	2.69	NS	NS
idhA	D-lactate dehydrogenase	NS	NS	4.68
hyaA	hydrogenase	−19.97	100.15	222.24
hyaB	hydrogenase	−12.06	103.30	346.63
frdA	fumarate reductase	−14.85	−5.18	NS
frdB	fumarate reductase	−10.49	NS	2.14
frdC	fumarate reductase	−10.21	NS	2.50
frdD	fumarate reductase	−7.06	NS	NS
hypA	Maturation of formate dehydrogenlyase	−11.77	NS	2.29
hypB	Maturation of formate dehydrogenlyase	−6.23	NS	4.48
hypC	Maturation of formate dehydrogenlyase	−5.72	NS	3.51
hypD	Maturation of formate dehydrogenlyase	−3.84	NS	4.26
hypE	Maturation of formate dehydrogenlyase	−2.05	NS	4.76
fdhF	formate dehydrogenase	2.18	−13.62	−3.08
hycA	formate hydrogenlyase	NS	94.92	90.19
hycB	formate hydrogenlyase	NS	35.49	1,656.22
hycC	formate hydrogenlyase	NS	30.91	83.73
hycD	formate hydrogenlyase	NS	33.74	216.46
hycE	formate hydrogenlyase	NS	30.57	93.44
hycF	formate hydrogenlyase	NS	35.73	784.84
hycG	formate hydrogenlyase	NS	10.03	18.65
hycH	formate hydrogenlyase	NS	12.16	18.28
hycI	formate hydrogenlyase	NS	4.63	8.73

NS, not statistically significant (FDR > 0.05).

### Transcriptional Changes in STEC Virulence Genes Commonly Used for Risk Assessments

The subset of genes shared between the STEC strains, but absent from the HS genome, includes known *E. coli* virulence factors. We examined selected virulence factors commonly used to assess human pathogenicity for differential expression in STEC upon adherence for 3 h to T84 IECs. STEC strain 97-3250 carries two Stx alleles, *stx1a* and *stx2a*, while 4865/96 carries only *stx2a*. The transcript containing the A subunit of *stx1a* in 97-3250 was not differentially expressed, while expression of the B subunit was downregulated 2.64-fold. For *stx2a*, neither subunit was differentially expressed in either of the STECs, which is not unexpected. The LEE PAI contains genes involved in intimate adherence to colonic epithelial cells as well as genes encoding effector proteins. All the genes located within the LEE PAI were downregulated in both STECs, with fold change values ranging from −10.91 (*ler*) to −75.25 (*escI*) for 97-3250 and −3.85 (*ler*) to −48.05 (*eae*) for 4865/96. In addition to virulence genes carried in the STEC chromosome, *ehxA*, catalase-peroxidase (*katP*), and an extracellular serine protease autotransporter (*espP*) are carried on a large virulence plasmid harbored in many pathogenic STEC strains. The *ehxA* gene was downregulated in both STEC strains, with fold changes of −5.32 and −2.16 in 97-3250 and 4865/96, respectively. The *katP* gene on the plasmid harbored in 97-3250 was not differentially expressed and 4865/96 does not carry *katP*. The *espP* gene was not differentially expressed in STEC 4865/96, while it was downregulated 3.91-fold in 97-3250.

### DEGs With No *E. coli* HS Homolog Potentially Involved in STEC Pathogenesis

In addition to the genes within the LEE PAI, the STEC strains shared 48 DEGs with no HS homolog. However, for six of the genes, the transcript was regulated in the opposite direction between the STEC strains, leaving 42 DEGs unique to the STEC strains with expression in both strains either increased or decreased upon 3 h adherence. To further corroborate that these 42 DEGs represent genes potentially contributing to pathogenesis in humans, we performed infection experiments utilizing polarized Caco-2 IECs, thereby reducing specific STEC-IEC and STEC-media type interaction effects. As for the transcriptomic experiments utilizing T84 IECs, DEGs were identified from a comparison of 3 h infections utilizing polarized Caco-2 IECs and STEC grown in planktonic culture. The resulting adhered *versus* planktonic transcriptional responses were compared to the T84 IEC results. Of the 42 DEGs, 20 genes were observed to be differentially expressed in both STECs and in the same direction as was observed in the T84 IEC experiments, with nine upregulated genes and 11 downregulated genes (**Table 4**). Protein sequences of the translated nucleotide sequences were used to verify or identify the genes utilizing BLASTp searches of the non-redundant protein sequence database at NCBI. Known virulence genes are included in the subset of genes, for example, *ehxA* and *perC*. Also included were the tellurite resistance genes *terZ, terA*, and *terB*, reported to also be involved in colicin resistance (Whelan et al., 1995) and increased resistance to oxidative stress caused by hydrogen peroxide (Valkova et al., 2007). There are two diacylglycerol kinase genes in both STECs, but only one copy in HS. The *dgkA* genes in the STEC strains with closest sequence homology to *dgkA* in HS were not differentially expressed; however, transcription of the second copy is reduced in the STECs (**Table 4**). Additionally, *lpxR*, a gene encoding a CAAX protease (Jandu et al., 2009), the P4 integrase gene, necessary for phage excision, and a gene with locus tag DA88_06695 encoding a protein with sequence homology to alpha/beta hydrolases but with unknown function, were expressed to a lesser extent in adhered compared to planktonic STEC cells. In contrast, other genes were upregulated after 3 h of infection. The immunoglobulin-binding regulator encoded by *ibrB* and a gene encoding colanic acid biosynthesis pyruvyl transferase, *wcaK*, demonstrated increased expression. A gene encoding a diguanylate cyclase was also upregulated, as well as *yhiM*, encoding an inner membrane protein with roles in acid resistance and in regulating growth of *E. coli* in conditions of low osmolarity and high temperature (Anderson et al., 2017). In addition, the gene identified as *yahM*, encoding an uncharacterized protein, was more highly transcribed. The IncFII plasmid replication initiator gene, *repA*, was also upregulated after 3 h of infection, however, *mobC*, involved in plasmid mobilization, was downregulated.

**Table 4:** Genes with no homolog in *E. coli* HS exhibiting differential expression in both STEC strains when adhered to both T84 and Caco-2 colonic epithelial cells compared to planktonic culture.

97-3250 Locus tag	4865/96 Locus tag	Gene	Function	FC 97-3250 T84	FC 97-3250 Caco-2	FC 4865/96 T84	FC 4865/96 Caco-2
DA88_01700	DC23_13485	***hyp1***	**hypothetical protein**	10.47	3.91	2.42	4.18
DA88_06695	DC23_04790		alpha/beta hydrolase, function unknown	−4.54	−7.45	−5.22	−4.86
DA88_11325	DC23_02285	***hyp2***	**hypothetical protein**	−5.97	−4.11	−2.29	−4.96
DA88_13870	DC23_23830	*dgkA*	diacylglycerol kinase	−3.83	−2.25	−2.22	−2.60
DA88_14020	DC23_10555	*terZ*	tellurite resistance	−5.87	−5.06	−4.99	−5.94
DA88_14025	DC23_10550	*terA*	tellurite resistance	−3.34	−4.75	−3.35	−4.40
DA88_14030	DC23_10545	*terB*	tellurite resistance	−4.17	−3.58	−2.14	−3.97
DA88_14145	DC23_03940	*ibrB*	immunoglobulin binding regulator	3.12	7.50	2.43	2.83
DA88_14150	DC23_03945	*perC*	transcriptional regulator	2.83	7.63	2.52	4.26
DA88_14155	DC23_03950	***hyp3***	**hypothetical protein**	2.54	3.65	3.31	2.93
DA88_14780	DC23_01755	*int*	bacteriophage P4 integrase	−2.62	−2.57	−2.12	−2.84
DA88_15545	DC23_10065	*wcaK*	colanic acid biosynthesis pyruvyl transferase	122.26	15.65	3.55	2.16
DA88_18555	DC23_22785	*yhiM*	environmental stress response	9.71	57.85	11.76	4.73
DA88_20255	DC23_11075	*yahM*	function unknown	2.52	8.11	3.72	9.13
DA88_26845	DC23_21615		CAAX protease	−13.35	−4.23	−5.82	−3.42
DA88_27945	DC23_14280		diguanylate cyclase	2.35	2.34	3.56	2.41
DA88_28745	DC23_18250	*ehxA*	enterohemolysin A	−5.32	−2.27	−2.16	−2.25
DA88_29555	DC23_13320	*mobC*	plasmid mobilization	−7.57	−4.73	−3.12	−2.64
DA88_30375	DC23_03830	*lpxR*	lipid A 3-O-deacylase	−9.22	−24.09	−35.96	−11.48
DA88_31020	DC23_01930	*repA*	IncFII plasmid replication initiation	2.13	3.26	2.24	5.15

Genes encoding potential novel virulence factors are shown in bold font.

### DEGs Representing Novel *E. coli* Virulence Genes

**Figure 4:**
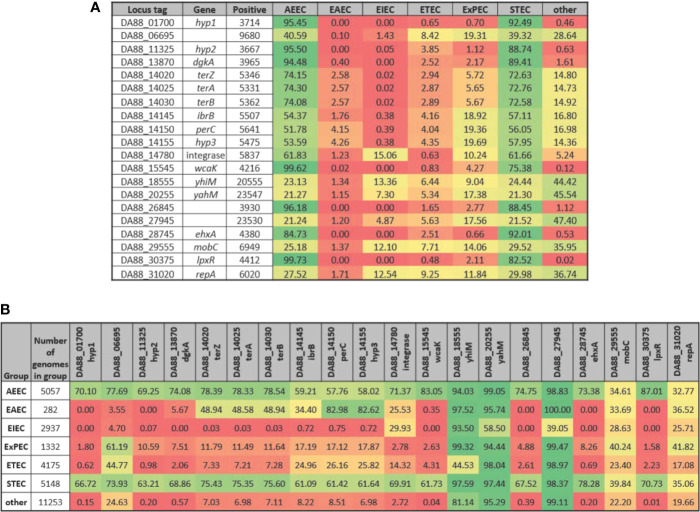
Presence of selected genes in *E. coli* genomes by pathotype. The STEC strains 97-3250 and 4865/96 were used in T84 and Caco-2 IEC infections and genes differentially expressed in infections for both IEC lines were determined. For each of the genes, the nucleotide sequences from 97-3250 were used in megablast queries performed on a database of 25,527 *E. coli* genomes. Percentages of **(A)** *E. coli* pathotypes comprising the positive matches and **(B)** genomes within a pathotype in which the gene is present were determined.

Along with genes of known or putative function that are differentially expressed in the same direction in both STEC strains upon adherence to both T84 and Caco-2 IECs, three of the DEGs represent hypothetical proteins (**Table 4**). These genes, referred to as *hyp1*, *hyp2*, and *hyp3*, are potentially novel virulence genes involved in pathogenesis in humans. However, the experiments undertaken in the present study include only two STEC strains and one commensal *E. coli* strain, thus, to substantiate the possibility that these genes may represent novel virulence genes, a total of 25,527 genomes were analyzed representing all known *E. coli* phylogroups as well as *Escherichia* cryptic lineages 1 and 6. To compare the presence of the three hypothetical genes in the different *E. coli* pathotypes with the presence of known virulence genes, all 20 genes in **Table 4** were included in the analysis. Of the 25,527 genomes, *hyp1*, *hyp2*, and *hyp3* were present in 3714, 3667, and 5475 *E. coli* genomes, respectively (**Figure 4A**). For the genomes possessing *hyp1* and *hyp2*, over 88% were classified as AEEC and/or STEC, while less than 1% of the genomes were classified as “other”. Note that a subset of STEC carry the LEE PAI, thus are also classified as AEEC, resulting in an overlap between the pathotypes as defined. The *hyp3* gene was present in a greater number of genomes and 58% were classified as STEC, while 14% of the genomes contain no pathotype-specific molecular markers. These results are consistent with those obtained for known virulence genes in the query set, however they do not demonstrate how widespread the three *hyp* genes are within the STEC pathotype. Thus, to further investigate, the percent of genomes of a given pathotype that are positive for each of the 20 genes was determined (**Figure 4B**). The results reveal that over 61% of STEC genomes carry at least one of the genes. In addition to STEC and AEEC, *hyp2* is carried by 10.6% of ExPEC genomes. The *hyp3* gene has a higher occurrence in a wider variety of pathotypes, notably in 82.6% of EAEC genomes. The *hyp1* and *hyp2* genes are almost exclusive to *E. coli* genomes that are classified as human pathogens, while *hyp3* was discovered in 7.0% of genomes carrying no molecular markers defining the genome as a known *E. coli* pathotype. The genomic location of the three hypothetical genes was examined using the closed STEC O26:H11 strain 11368 genome (accession #AP010953.1) as a reference strain. Analysis revealed two of the genes to be carried on prophages. The *hyp1* gene has homolog ECO26_2650 and is within prophage ECO26_P14 inserted at *ryeB*, and the *hyp2* gene has homolog ECO26_1103 and is within prophage ECO26_P03 inserted at *yccA*. The other gene, *hyp3*, with homolog ECO26_1340, is located in the tellurite adherence island (TAI) ECO26_IE02 inserted at *serX*. The sequences of the contigs containing the three *hyp* genes in the draft genomes of 97-3250 and 4865/96 are consistent with the *hyp* genes having locations within these mobile genetic elements.

### Cytokine and Chemokine Expression in Polarized IECs

**Figure 5:**
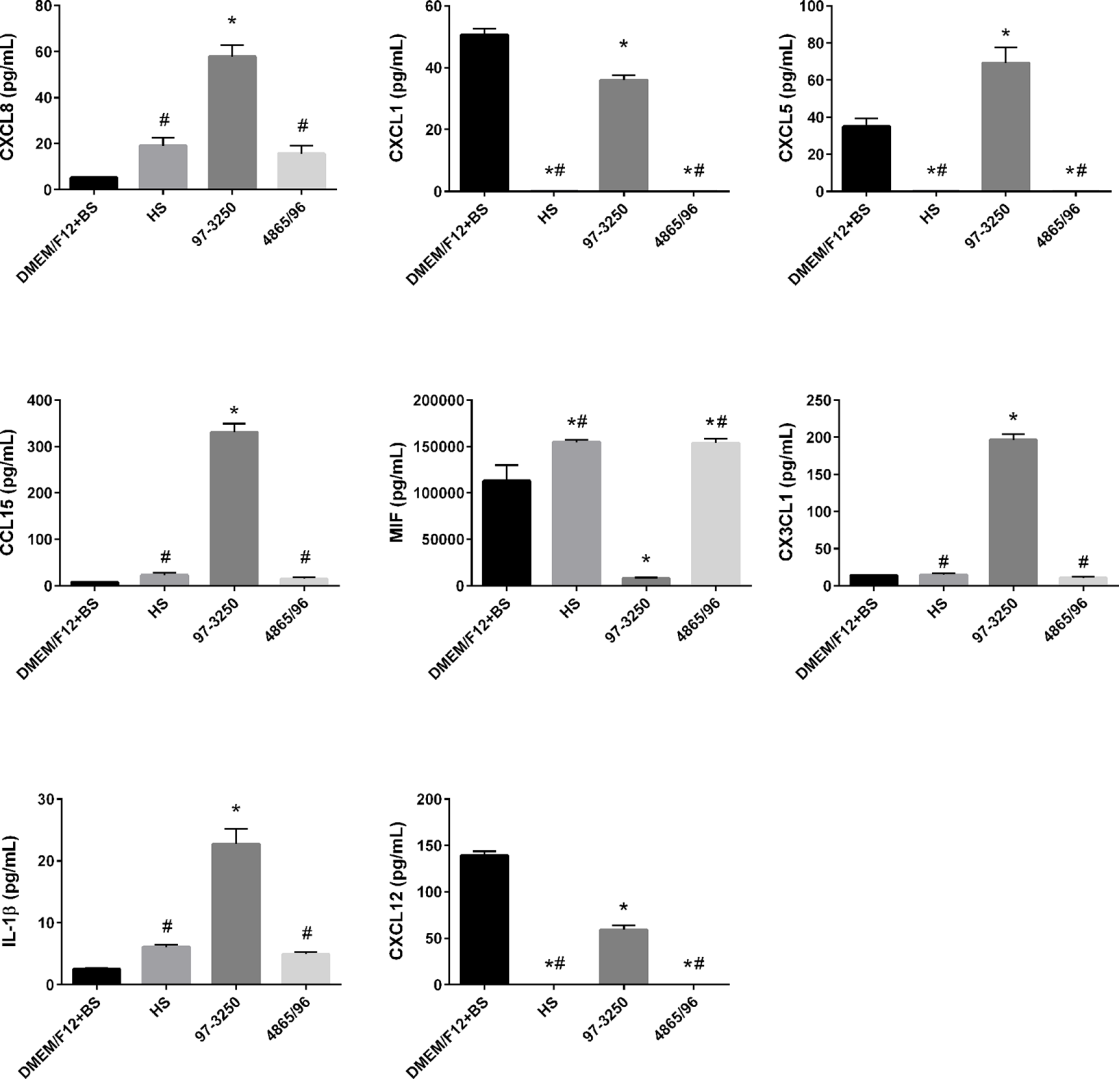
Secretion of PMN- and Monocyte-specific cytokines by polarized T84 cells. Polarized T84 cells were uninfected (DMEM/F12 + BS) or apically infected with commensal *E. coli* strain HS, or STEC strains 97-3250 or 4865/96 at a MOI of 100. Basolateral supernatants were collected after 3 h and analyzed using a human cytokine multiplex assay. Data are presented as a mean value of each cytokine ± standard error of the mean (n = 3). An asterisk (*) denotes significant differences from uninfected controls (*p* value < 0.01). A number sign (#) denotes significant differences between 97-3250 and either HS or 4865/96 infections (*p* value < 0.0001).

**Figure 6:**
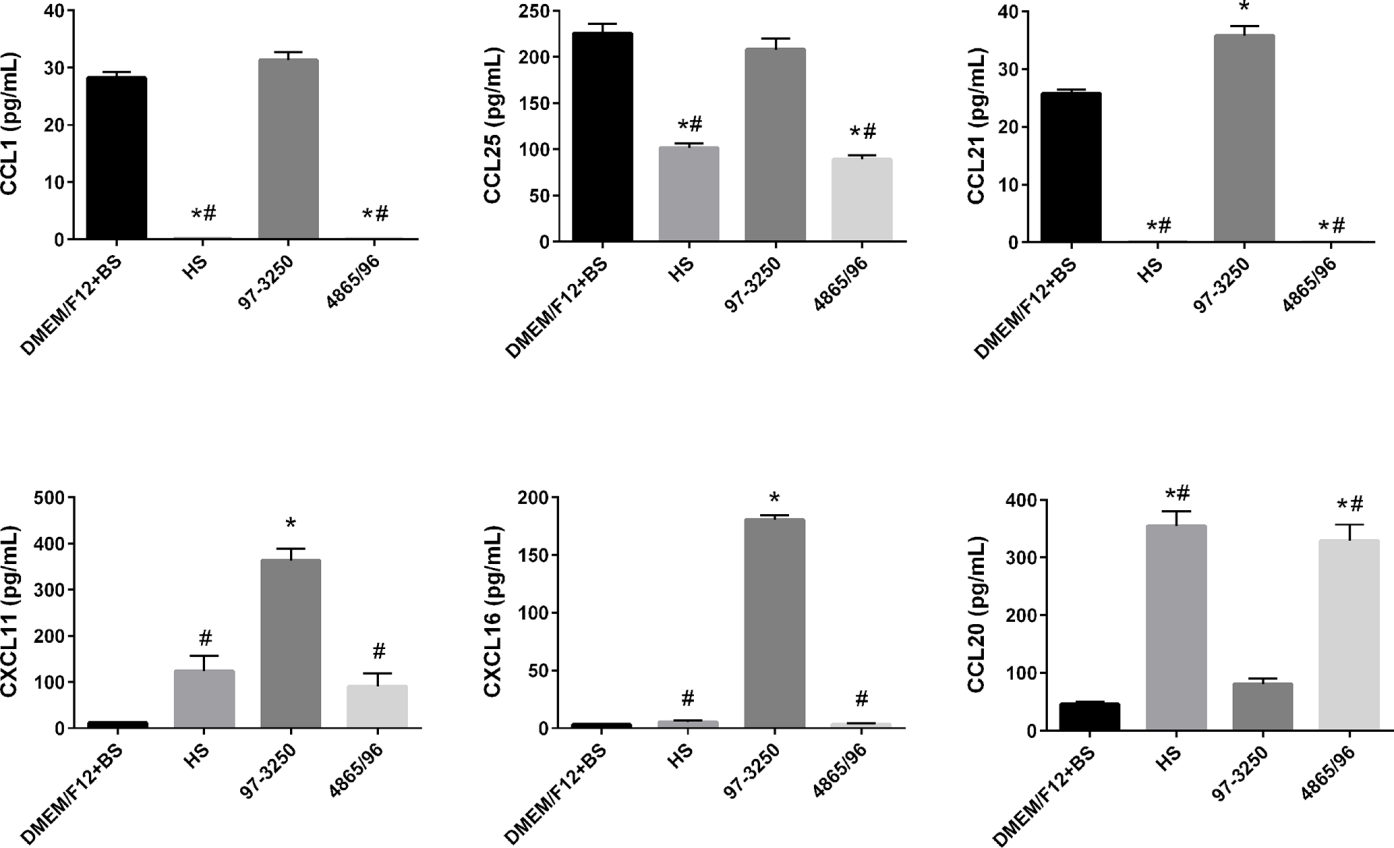
Secretion of T cell- and DC-specific cytokines by polarized T84 cells. Polarized T84 cells were uninfected (DMEM/F12 + BS) or apically infected with commensal *E. coli* strain HS, or STEC strains 97-3250 or 4865/96 at a MOI of 100. Basolateral supernatants were collected after 3 h and analyzed using a human cytokine multiplex assay. Data are presented as a mean value of each cytokine ± standard error of the mean (n = 3). An asterisk (*) denotes significant differences from uninfected controls (*p* value < 0.01). A number sign (#) denotes significant differences between 97-3250 and either HS or 4865/96 infections (*p* value < 0.0001).

To determine whether the polarized IEC line T84 is a good model to screen for pathogenicity of individual non-O157 STEC strains, basolateral supernatants from uninfected T84 cells and T84 cells infected with HS, 97-3250, or 4865/96 were analyzed for cytokine biomarker expression using a multiplex assay of 40 cytokines (**Table S1**). Cytokine data sets having fewer than two data points or where the highest concentrations were less than 10 pg/ml, were excluded from further analyses. The remaining cytokine profiles (24 data sets) were compared for differences between uninfected and infected T84 cells as well as differences between IECs infected with HS and the STEC strains. In almost half of the cytokine profiles analyzed, infection with HS, 97-3250, or 4865/96 resulted in significantly lower levels of several cytokines (CCL27, CCL11, CXCL6, IL-10, IL-16, CCL7, CXCL9, CCL23, CCL17, & CCL19) compared to uninfected cells, and these cytokine levels were no greater than 20 pg/mL in infected cells (data not shown), except for CXCL1 and CXCL12 (**Figure 5**). For cytokine profiles CCL1, CCL20, and CCL25, the levels from uninfected T84 cells and those infected with 97-3250 were not significantly different (**Figure 6**). Similarly, the cytokine levels produced by T84 cells infected with HS and 4865/96 did not differ significantly. However, the cytokine levels from both uninfected and 97-3250-infected T84 cells were significantly different from both HS- and 4865/96-infected T84 cells (**Figure 6**). Only two cytokine profiles, CCL21 and CXCL5, demonstrated a pattern where cytokine levels produced by uninfected T84 cells were significantly higher than those infected with HS or 4865/96, but significantly lower than T84 cells infected with 97-3250 (**Figures 5**, **6**). Conversely, for only one cytokine, MIF, a pattern was observed where cytokine levels in uninfected T84 cells were significantly lower than those infected with HS and 4865/96, but significantly higher than those infected with 97-3250 (**Figure 5**). In the remaining six cytokine profiles (CX3CL1, IL-1*β*, CXCL8, CXCL11, CCL15, and CXCL16), the cytokine levels from T84 cells infected with 97-3250 were significantly higher than levels from uninfected T84 cells and those infected with HS or 4865/96 (**Figures 5**, **6**). Furthermore, the cytokine levels from the uninfected T84 cells and T84 cells infected with HS or 4865/96 were not significantly different from each other and were expressed at relatively low levels compared to cells infected with 97-3250 (**Figures 5**, **6**).

## Discussion

### Expression Comparisons Between the STECs and Commensal *E. coli* HS

Interestingly, despite a larger gene repertoire, transcriptomic analysis demonstrated that the pathogenic STEC strain 4865/96 had a significantly lower percentage of DEGs than the commensal *E. coli* HS in transitioning between planktonic and adhered conditions, whereas STEC 97-3250 had a greater percentage (**Table 1**, **Figure 1A**). While we observed considerable differences in global transcriptional responses among the three strains as evidenced by the number of genes that were differentially expressed in only one or two strains (**Figure 1B**), the DEGs were classified similarly overall by GO analysis into categories that would be expected when transitioning from a planktonic to adhered lifestyle (**Figures 2A–C**). The Pho regulon includes a large network of genes associated with many metabolic processes and adaptive responses. In addition, numerous studies have demonstrated that the Pho regulon is linked to a more complex network of genes influencing bacterial pathogenesis including the LEE genes and other virulence attributes (Cheng et al., 2009; Crepin et al., 2011; Chekabab et al., 2014; Santos-Beneit, 2015). The disparate transcriptional responses of *phoBR* and *pstSCAB* between the STECs and HS (**Table 2**) may indicate that the Pho regulon plays a critical role in STEC survival and colonization in the human gut.

Metabolic pathways are a category where our RNA-Seq results demonstrated significant diversity among the three *E. coli* strains (**Table 3**). Metabolic diversity and/or shifts in metabolism during infection and successful colonization among STEC strains and among other *E. coli* pathotypes have been observed in various *in vitro* and *in vivo* studies (Alteri et al., 2009; Kim et al., 2009; Maltby et al., 2013; Pieper et al., 2013; Hazen et al., 2015; Schinner et al., 2015; Chen et al., 2020). Additional studies demonstrate that some Stx2a phages impact carbon source utilization by *E. coli* (Berger et al., 2019), and that PerC in EPEC also affects transcription of genes involved in metabolism (Mellies et al., 2017). The STEC strains utilized in our study carry multiple phages and *perC* homologs, cluster with different phylogroups, and have unique gene content, which taken together create the possibility of transcriptional responses of various genes contributing in ways that result in individual carbon usage patterns. Greater *E. coli* pathogenicity may not be associated with a particular pattern of metabolism, rather, metabolic diversity compared to resident commensal *E. coli* and other microflora would be advantageous and may allow an STEC strain with a unique carbon and nitrogen utilization pattern to gain a foothold and colonize the human colon. Our results are consistent with the idea that the two STEC strains used in our study employ differing metabolic transcriptional strategies for host infection.

Amino acids can serve as sources of carbon and nitrogen along with their role in protein synthesis. Arginine, in particular, plays additional vital roles in bacterial cell survival, such as its use in imparting acid resistance (Kanjee and Houry, 2013; Charlier and Bervoets, 2019) and as a substrate for the synthesis of the polyamines putrescine and spermidine (Igarashi and Kashiwagi, 2018; Charlier and Bervoets, 2019). With the increased transcription of arginine biosynthesis genes along with uptake of extracellular arginine demonstrated in our RNA-Seq results (**Table 2**, **Figure 3**), the STECs possess a greater quantity of arginine in the adhered state versus planktonic conditions than HS. Arginine can be used by *E. coli* for the synthesis of polyamines, and polyamines have been shown to increase general protein synthesis and in particular to stimulate the translation of a specific set of proteins involved in increasing cell viability and biofilm formation during stationary phase in *E. coli* (Igarashi and Kashiwagi, 2018). Extracellular arginine can be utilized by IECs to either synthesize polyamines, which are vital for IEC proliferation, or to generate nitric oxide (NO), a host defense against pathogens. While some pathogens modulate the quantity of NO produced by host cells either by expressing an arginase gene encoding an enzyme used in the pathway for polyamine biosynthesis from the substrate arginine, or by upregulating host cell arginase (Das et al., 2010), previous studies have demonstrated that the extracellular intestinal pathogen *Giardia* decreases NO production by IECs by actively importing arginine, thus reducing the arginine available to the IECs (Eckmann et al., 2000; Stadelmann et al., 2012; Stadelmann et al., 2013). This competition for arginine between the pathogen and IECs also reduces the arginine available for polyamine biosynthesis by the IECs, leading to reduced IEC turnover which may aid in pathogen colonization (Stadelmann et al., 2012). Our results demonstrate that in transitioning from planktonic growth to IEC adherence, the STEC strains scavenge both arginine and polyamines to a greater extent than HS, which downregulated their import (**Table 2**). Limiting the generation of NO by IECs serves yet another purpose in addition to cell survival for STEC. Inhibition of *stx2* gene expression and phage synthesis by NO has been previously demonstrated (Vareille et al., 2007), thus attenuation of NO production by decreasing arginine availability to IECs affects not only the ability of STEC to colonize and survive, but also affects virulence.

Tryptophan is another important amino acid involved in metabolic cross talk between host and pathogen (Ren et al., 2018). Depletion of tryptophan available to IECs has been demonstrated to lessen the host immune response (Kong et al., 2018; Ren et al., 2018). When transitioning from a planktonic to adhered lifestyle, the gene encoding the tryptophan importer Mtr was upregulated in the STEC strains while considerably downregulated in HS (**Table 2**), suggesting the STECs actively compete with IECs for tryptophan in the adhered state to a much greater extent than HS. Tryptophanase, encoded by *tnaA* and decreased in transcription in the STECs (**Table 2**), catabolizes tryptophan to indole, ammonia, and pyruvate which allows *E. coli* to utilize tryptophan as a source of carbon and nitrogen in addition to using it in protein synthesis (Yanofsky et al., 1991). Indole facilitates inter- and intracellular signaling among microbiota in the human intestine, and indole concentration affects expression of genes associated with colonization and virulence in pathogenic *E. coli* including the LEE genes and *stx2a* (Lee and Lee, 2010; Bommarius et al., 2013; Platenkamp and Mellies, 2018; Kumar and Sperandio, 2019). The pronounced disparity between the STECs and HS in the regulation of genes associated with tryptophan biosynthesis, import, and catabolism when transitioning to an adhered lifestyle will result in differing effects on the interplay between the human host and *E. coli* cells.

The DppBCDF permease, in conjunction with the DppA periplasmic substrate-binding protein, actively transports dipeptides across the inner cell membrane. *In vivo* and *in vitro* experiments have demonstrated the importance of *dppA* in UPEC infection of the bladder and kidneys (Alteri et al., 2009), and our results suggest the *dppBCDF-dppA* genes are also important in STEC infection of IECs. Interestingly, DppBCDF-DppA has been identified to function as an inner membrane heme transporter in *E. coli* (Letoffe et al., 2006), and an outer membrane heme receptor, ChuA, was identified in an STEC O157:H7 strain (Torres and Payne, 1997). The *chuA* gene is found more frequently in STEC isolates of serotypes typically associated with severe disease including O145 strains, and heme uptake is thought to be advantageous for colonization and contribute to pathogenicity in STEC (Bosilevac and Koohmaraie, 2011). Of the three *E. coli* strains included in this study, 4865/96 is the only strain carrying *chuA* and transcription is upregulated 10.39-fold after 3 h of infection. Despite lacking *chuA*, many O26 strains have been observed to utilize exogenous heme, suggesting an alternate gene for transport of heme through the outer membrane (Kresse et al., 2007). The *hma* gene was identified in UPEC as encoding an alternate heme receptor (Hagan and Mobley, 2009), but none of the *E. coli* strains utilized in our study carry a *hma* homolog. However, this does not preclude the presence of an as yet unidentified heme receptor. The *dpp* genes are downregulated to a lesser extent in the STECs compared to HS after IEC infection (**Table 2**), and this response may be due to a greater need for dipeptide transport in the STECs upon adherence compared to HS, but heme transport offers an alternate or additional explanation.

### Expression of STEC Virulence Genes

The RNA-Seq results in this study are in agreement with many previous studies that demonstrate the importance of the LEE PAI and *ehxA* to STEC pathogenicity. The presence/absence patterns observed from examining 25,527 *E. coli* genomes for the three hypothetical DEGs identified as possible novel virulence genes are comparable to those of other known virulence genes, substantiating their identification as factors involved in STEC pathogenicity (**Figure 4**). Furthermore, their location in mobile genetic elements is consistent with identification as virulence genes. Collectively, our results indicate that these three genes represent novel *E. coli* virulence factors. Given that *hyp2* is downregulated in adhered compared to planktonic STEC cells, the product of the *hyp2* gene is likely important for survival and initial adherence in the human gastrointestinal tract, while the products of *hyp1* and *hyp3* would be predicted to be important for maintaining colonization after initial adherence.

The transcriptomics results in this study demonstrate not only the transcriptional regulation of commonly cited virulence factors but also highlight the fact that STEC utilizes other defense mechanisms to initially establish infection in the human host. The product of the gene encoded in 97-3250 and 4865/96 by locus tags DA88_26845 and DC23_21615, respectively, has been previously identified as a CAAX protease and potential virulence factor involved in subversion of IFN*γ*-Jak1,2-STAT-1 signaling (Jandu et al., 2009). Our results corroborate the finding that this protease is a virulence factor. The lipid A portion of lipopolysaccharide (LPS) is recognized by the host innate immune system resulting in production of pro-inflammatory cytokines. In an attempt to evade detection by the host, *Helicobacter pylori, Yersinia enterocolitica, Vibrio cholerae, Salmonella*, and many STEC strains remodel the lipid A structure, thereby attenuating the host inflammatory response (Reynolds et al., 2006; Cullen et al., 2011; Ogawa et al., 2018). Modification of lipid A is accomplished utilizing the 3'-O-deacylase encoded by *lpxR*. Expression of *lpxR* is regulated by Ler and Pch and is part of the virulence regulon including the LEE genes (Ogawa et al., 2018), thus the downregulation of both the LEE PAI and *lpxR* demonstrated in our work is internally consistent. Diacylglycerol kinase, encoded by *dgkA*, is a small enzyme spanning the inner cell membrane that plays a role in lipid recycling during membrane-derived oligosaccharide biosynthesis and in the recycling of diacylglycerol produced during LPS biosynthesis (Van Horn and Sanders, 2012). The second copy of *dgkA*, carried by the STECs but having less sequence homology to the *dgkA* gene found in HS, was downregulated in the STEC strains. An interrogation of 25,527 *E. coli* genomes demonstrated that this copy of *dgkA* is exclusive to pathogenic *E. coli*, predominantly STEC or AEEC (**Figure 4B**). Whether the second *dgkA* gene has the same physiological function as the *dgkA* found in all *E. coli* genomes is unknown. Finally, both the P4 integrase gene and *mobC* were expressed more highly in planktonic STEC cells than after adherence to IECs for 3 h, suggesting a higher rate of transfer of some mobile genetic elements prior to establishment of infection.

In contrast to virulence factors more highly expressed before adherence to IECs, transcription of other virulence factors was increased after 3 h of infection (**Table 4**). These included the transcriptional regulators *perC* and *ibrB*. The role of *perC* in virulence and niche adaptation has been well described (Mellies et al., 2017). In comparison to strains lacking *ibrB*, under inducing conditions the presence of *ibrB* in the genome activates enhanced expression of Eib on the bacterial cell surface (Sandt et al., 2002). The gene encoding IbrB is not restricted genomically to STEC but, similar to the *terZAB* and *perC* genes, is found in subsets of other *E. coli* pathotypes as well as approximately 8% of the *E. coli* genomes in our analysis carrying no pathotype-specific markers (**Figure 4B**). Transcription of the gene *wcaK* in the colanic acid biosynthesis pathway was also upregulated in the STECs after adherence to IECs (**Table 4**). Colanic acid is a secreted exopolysaccharide establishing a capsule around the bacterial cell that is protective from a variety of environmental stresses and plays a role in biofilm formation (Mao et al., 2001; Kocharunchitt et al., 2014; Miajlovic et al., 2014). Although the colanic acid capsule is produced and confers protection in the environment outside the human host, studies have demonstrated that it is protective against the bactericidal effects of serum in extraintestinal *E. coli* pathogens (Miajlovic et al., 2014; Ma et al., 2018). Interestingly, although even commensal *E. coli* can produce colanic acid, the sequence of *wcaK* carried in the STECs used in our work is fairly restricted to STEC and other AEEC genomes (**Figure 4B**). Whether this conveys any physiological relevance is unknown. Finally, there were genes found in most *E. coli* irrespective of pathotype, but not carried by HS, that were determined to be important for colonization, namely *yhiM*, *yahM*, and a diguanylate cyclase gene (**Table 4**, **Figure 4B**). *E. coli* genomes carry a variety of diguanylate cyclase genes. Diguanylate cyclase is involved in the synthesis of cyclic-di-GMP, a second messenger signaling molecule that regulates various physiological functions involved in the transition from a planktonic to sessile state (Hengge, 2009; Whiteley and Lee, 2015). Consistent with our work, several diguanylate cyclase genes were identified as DEGs in transcriptomic experiments comparing planktonic ETEC and ETEC adhered to Caco-2 IECs (Kansal et al., 2013). Additionally, reduced intracellular levels of cyclic-di-GMP have been demonstrated to reduce adherence of STEC O157:H7 to HT-29 epithelial cells and cattle colon explants (Hu et al., 2013).

### Cytokine/Chemokine Responses from IECs

We observed the differential induction of a range of cytokines/chemokines following infection of polarized T84 IECs with pathogenic STEC or commensal *E. coli*. The PMN chemoattractants CXCL8, CXCL1, CXCL5, and CCL15 were differentially expressed in T84 cells infected with 97-3250 compared to HS or 4865/96 (**Figure 5**). While the role of epithelial-derived CXCL8/IL-8 in mucosal defense and inflammatory responses related to STECs may not be fully understood, its role in PMN recruitment during acute inflammation is considered an important early component of host defense against bacterial pathogens. Numerous studies have reported the involvement of CXCL8/IL-8 in STEC pathogenesis including intestinal barrier disruption through the induction of PMN transmigration into the intestinal lumen, leading to fecal leukocytes in infected patients, Stx access to the bloodstream, and PMN transport of Stxs to target cells and tissues (Elliott et al., 1994; Slutsker et al., 1997; Hurley et al., 2001; Karve et al., 2017). Several STEC factors such as Stxs, flagellin, LPF, and HCP can induce CXCL8/IL-8, CXCL1/GRO-α, and CXCL5/ENA-78 in a variety of intestinal epithelial cell lines (Yamasaki et al., 1999; Thorpe et al., 2001; Berin et al., 2002; Rogers et al., 2003; Miyamoto et al., 2006; Ledesma et al., 2010; Farfan et al., 2013). Our results demonstrate that 97-3250 may promote greater PMN infiltration during IEC infection than 4865/96 or HS.

In correlation with our PMN chemoattractant results, expression patterns of the monocyte/macrophage cytokines/chemokines macrophage migration inhibitory factor (MIF), CX3CL1/Fractalkine, IL-1β, and CXCL12/SDF-1 differed significantly between T84 cells infected with 97-3250 *versus* 4865/96 and HS (**Figure 5**). MIF is constitutively produced by untreated human IECs *in vitro* and *in vivo*, biologically active, and inhibits the migration of macrophages when there is no infection (Maaser et al., 2002). In our model, the reduced MIF levels induced by 97-3250 and increased MIF levels induced by 4865/96 and HS suggest that 97-3250 promotes while 4865/96 and HS reduce macrophage recruitment by IECs. IL-1β has been reported to modulate various aspects of immune function including the increased expression of adhesion molecules that can promote monocyte/macrophage and neutrophil infiltration to infection sites as well as the induction of CX3CL1/Fractalkine by IECs (Huang et al., 1996; Muehlhoefer et al., 2000; Dinarello, 2009). Recruited or circulating macrophages that encounter Stxs may produce additional cytokines, including IL-1β, leading to increased inflammation and disease (Ramegowda and Tesh, 1996; Harrison et al., 2004; Lee et al., 2013; Lee et al., 2016). Our IL-1β and CX3CL1/Fractalkine results (**Figure 5**) demonstrate that 97-3250 may promote a greater inflammatory response than 4865/96 through macrophage recruitment. In the intestine, CXCL12/SDF-1 may play a role in immune surveillance through the promotion of monocyte extravasation (Bleul et al., 1996). Although reduced compared to uninfected T84 cells, CXCL12/SDF-1 is still induced during 97-3250 infection and may allow for normal immune surveillance, while its absence during 4865/96 and HS infections may limit surveillance functions (**Figure 5**). Collectively, our monocyte/macrophage chemoattractant expression profiles suggest that 97-3250 infections may promote monocyte/macrophage infiltration to the intestine, and thus inflammation, while HS and 4865/96 may downregulate or fail to promote these responses.

The role of T cells and dendritic cells (DCs) in STEC pathogenesis in humans is not well understood or studied. Interestingly, we observed significantly higher levels of the T cell chemoattractants CX3CL1, CCL25, CCL1, CCL21, CXCL11, and CXCL16 but lower levels of the DC chemoattractant CCL20/MIP-3*α* in T84 cells infected with 97-3250 compared to cells infected with HS or 4865/96 (**Figure 6**). The role of these T cell chemoattractants in STEC pathogenesis is unknown; however, CCL25 (Wurbel et al., 2011; Trivedi et al., 2016; Hernandez-Ruiz and Zlotnik, 2017), CCL1 (Regan et al., 2013), CCL21 (Luther et al., 2002), CXCL11 (Dwinell et al., 2001), and CXCL16 (Diegelmann et al., 2010) have established roles in the maintenance of normal intestinal functions or during intestinal inflammation. The differential expression of these T cell chemoattractants observed during infection with HS or 4865/96 compared to 97-3250 suggests that 4865/96 may be recognized as a nonpathogenic *E. coli* strain, possibly as a way to evade detection. CCL20/MIP-3*α* can be expressed in inflamed epithelial crypts and at varying levels in IECs depending on the STEC serotype and their TTSS and flagellin (Dieu et al., 1998; Gobert et al., 2008). The differing CCL20/MIP-3*α* levels induced by 97-3250 and 4865/96 in our study are supported by these previous reports and further suggest that these two STEC serotypes are able to elicit opposing inflammatory responses on their path to causing disease.

## Summary

*In vitro* infection models do not fully represent the overall *in vivo* condition, which includes a complex interplay between host cells, resident microbiota, and the invading STEC. Nevertheless, the cytokine and transcriptomics results presented in this study provide further insight into similarities and differences elicited by pathogenic STEC, a commensal *E. coli* strain, and IECs at the onset of infection. Although our infection model was not able to provide a single distinct cytokine profile indicative of STEC infection resulting in severe clinical outcome, it is a useful model for understanding individual cellular activities in response to STEC and highlights the variable strain-specific responses to pathogenic STEC that arise irrespective of host differences. Overall, our cytokine profiles suggest that 4865/96 either inhibits the proinflammatory response of polarized T84 IECs to resemble nonpathogenic *E. coli* commensals like HS, or lacks certain factors, which may be present in 97-3250, that elicit a proinflammatory response. While the transcriptomics results demonstrate biochemical pathways, regulons, and shared virulence genes that are regulated similarly in the STECs, STEC genomes contain a considerable mobilome that is often diverse and this is demonstrated by the number of unique genes found in the STECs used in this study (**Figure 1A**). Moreover, not all shared genes are transcriptionally regulated in an analogous way. These differences in STEC gene content and/or regulation support both the disparate IEC cytokine responses and DEGs associated with metabolism observed in this study and suggest there are multiple pathways involving different sets of genes that may lead to successful infection. However, through our focus on genes shared between the STECs, we have identified candidate genes likely representing novel STEC virulence factors that, if proven by further phenotypic study, may be added to the repertoire of genes considered for risk assessments.

## Data Availability Statement

Sequence reads were deposited in the NCBI Sequence Read Archive (SRA) under the BioProject number PRJNA641161.

## Author Contributions

LH and SL conceived of and designed the project and conducted experiments. SL performed the RNA-Seq analysis, LH analyzed the cytokine data, and DL and MM performed other analyses. LH and SL wrote the manuscript. All authors contributed to the article and approved the submitted version.

## Conflict of Interest

The authors declare that the research was conducted in the absence of any commercial or financial relationships that could be construed as a potential conflict of interest.

## Supplementary Material

Supplementary Table 1 Panel of cytokines/chemokines included in the assay. *Data sets with fewer than 2 data points or where the highest concentrations were less than 10 pg/ml were excluded.
