# Supplementary material for: Comparative Transcriptomics of Shiga Toxin-Producing and Commensal Escherichia coli and Cytokine Responses in Colonic Epithelial Cell Culture Infections
Source: Front Cell Infect Microbiol. 2020 Oct 26;10:575630. doi: 10.3389/fcimb.2020.575630 (PMC7649339; doi:10.3389/fcimb.2020.575630)
Supplement: Supplementary Table 1 — Panel of cytokines/chemokines included in the assay. *Data sets with fewer than 2 data points or where the highest concentrations were less than 10 pg/ml were excluded. [file Table_1.docx]

**Table S1. Panel of cytokines/chemokines included in the assay**

| **Cytokine/chemokine** | **Included in analyses*** |
| --- | --- |
| CCL21/6Ckine | Y |
| CXCL13/BCA-1 | N |
| CCL27/CTACK | Y |
| CXCL5/ENA-78 | Y |
| CCL11/Eotaxin | Y |
| CCL24/Eotaxin-2 | N |
| CCL26/Eotaxin-3 | N |
| CX3CL1/Fracktalkine | Y |
| CXCL6/GCP-2 | Y |
| GM-CSF | N |
| CXCL1/Gro-α | Y |
| CXCL2/Gro-β | N |
| CCL1/I-309 | Y |
| IFN-γ | N |
| IL-1β | Y |
| IL-2 | N |
| IL-4 | N |
| IL-6 | N |
| CXCL8/IL-8 | Y |
| IL-10 | Y |
| IL-16 | Y |
| CXCL10/IP-10 | N |
| CXCL11/I-TAC | Y |
| CCL2/MCP-1 | N |
| CCL8/MCP-2 | N |
| CCL7/MCP-3 | Y |
| CCL13/MCP-4 | N |
| CCL22/MDC | N |
| MIF | Y |
| CXCL9/MIG | Y |
| CCL3/MIP-1α | N |
| CCL15/MIP-1δ | Y |
| CCL20/MIP-3α | Y |
| CCL19/MIP-3β | Y |
| CCL23/MPIF-1 | Y |
| CXCL16/SCYB16 | Y |
| CXCL12/SDF-1α+β | Y |
| CCL17/TARC | Y |
| CCL25/TECK | Y |
| TNF-α | N |

*Data sets with fewer than 2 data points or where the highest

concentrations were less than 10 pg/mL were excluded
